# Lung tumor motion change during stereotactic body radiotherapy (SBRT): an evaluation using MRI

**DOI:** 10.1120/jacmp.v15i3.4434

**Published:** 2014-05-08

**Authors:** Anneyuko I. Saito, Kenneth R. Olivier, Jonathan G. Li, Chihray Liu, Heather E. Newlin, Ilona Schmalfuss, Shinsuke Kyogoku, James F. Dempsey

**Affiliations:** ^1^ Department of Radiology Juntendo Urayasu Hospital Urayasu Japan; ^2^ Department of Radiation Oncology Mayo Clinic Rochester MI USA; ^3^ Department of Radiation Oncology University of Florida College of Medicine Gainesville FL USA; ^4^ Department of Radiology University of Florida College of Medicine Gainesville FL USA; ^5^ ViewRay Inc. Cleveland OH USA

**Keywords:** lung cancer, MRI, SBRT, cone‐beam CT

## Abstract

The purpose of this study is to investigate changes in lung tumor internal target volume during stereotactic body radiotherapy treatment (SBRT) using magnetic resonance imaging (MRI). Ten lung cancer patients (13 tumors) undergoing SBRT (48 Gy over four consecutive days) were evaluated. Each patient underwent three lung MRI evaluations: before SBRT (MRI‐1), after fraction 3 of SBRT (MRI‐3), and three months after completion of SBRT (MRI‐3m). Each MRI consisted of T1‐weighted images in axial plane through the entire lung. A cone‐beam CT (CBCT) was taken before each fraction. On MRI and CBCT taken before fractions 1 and 3, gross tumor volume (GTV) was contoured and differences between the two volumes were compared. Median tumor size on CBCT before fractions 1 (CBCT‐1) and 3 (CBCT‐3) was 8.68 and 11.10 cm^3^, respectively. In 12 tumors, the GTV was larger on CBCT‐3 compared to CBCT‐1 (median enlargement, 1.56 cm^3^). Median tumor size on MRI‐1, MRI‐3, and MRI‐3m was 7.91, 11.60, and 3.33 cm^3^, respectively. In all patients, the GTV was larger on MRI‐3 compared to MRI‐1 (median enlargement, 1.54 cm^3^). In all patients, GTV was smaller on MRI‐3m compared to MRI‐1 (median shrinkage, 5.44 cm^3^). On CBCT and MRI, all patients showed enlargement of the GTV during the treatment week of SBRT, except for one patient who showed minimal shrinkage (0.86 cm^3^). Changes in tumor volume are unpredictable; therefore, motion and breathing must be taken into account during treatment planning, and image‐guided methods should be used, when treating with large fraction sizes.

PACS number: 87.53.Ly

## I. INTRODUCTION

Stereotactic body radiotherapy (SBRT) was initially introduced as an effective treatment method for early primary lung cancers only.^(1)^ However, it is now widely used for treatment of other anatomical sites including primary and metastatic tumors in the liver, spine, pancreas, kidney, and prostate, as well as metastatic tumors to the lung.^(2)^ For treatment of primary and metastatic lung cancers, several fraction schedules are available including 50 to 70 Gy in 10 fractions, 50 to 60 Gy in 8 fractions, 45 to 55 Gy in 5 fractions, 44 to 50 Gy in 4 fractions, and 54 to 66 Gy in 3 fractions.^(3)^ The time between treatment planning to delivery is commonly one to two weeks, independent of the type of fractionation schedule used. Radiotherapy volumes and fields derived from the initial planning computed tomography (CT) images are typically used throughout the treatment without any modifications. Tumors, however, may change in size and location between planning and treatment or between each treatment. Breathing patterns may also alter, or the pattern recorded at simulation may not reflect the pattern seen at treatment. Such changes in size and motion can affect the internal target volume (ITV), thereby affecting the accuracy of the SBRT. The aim of this study was to investigate changes in lung tumor ITV during SBRT using magnetic resonance imaging (MRI) and cone‐beam CT (CBCT).

## II. MATERIALS AND METHODS

### A. Patients

From March 2006 to March 2007, a total of 13 lesions in ten patients were treated with SBRT in consecutive daily fractions using daily image guidance with CBCT for medically inoperable non‐small cell lung cancer or metastatic lung lesions at our institution. The patient characteristics are shown in Table 1.

**Table 1:** Patient characteristics (10 patients, 13 tumors)

*Characteristics*	*Value*
Age (years)	76 (range, 44–84)
Gender (Male:Female)	3:7
Tumor Location	
Right upper lobe	7
Right lower lobe	2
Left upper lobe	4
Pathology	
Primary NSCLC	7
Metastatic lung cancer	3
No pathology obtained	3
Median tumor size^a^ (ml)	74 (range, 39–105)

a Before the start of radiotherapy.

NSCLE=non−small cell lung cancer.

### B. Simulation and treatment

All patients underwent simulation and treatment planning using a four‐dimensional CT to assess respiratory motion (Philips Brilliance, Philips Healthcare, Andover, MA). Patients were simulated while free breathing (KV=120,mAs=250/slice), and 4D lung (KV=120,mAs=600/slice). The image guidance we employed with CBCT has been described elsewhere.^(4)^ In summary, we took a CBCT image (120 KV, 40 mA, 40 mS or 80 mA for large patients) before every single fraction of SBRT. The SBRT prescription was 48 Gy delivered in four consecutive days.

### C. Measurement

Each patient underwent three MRI (open 0.2 T MAGNETOM Siemens, v. B33G; Siemens Healthcare, Erlangen, Germany) evaluations of the lung. The first was taken before SBRT (median, one day; range, up to four days, before SBRT), a second after treatment on the third day of SBRT, and the third MRI at three months after completing SBRT. During each MRI, axial T1‐weighted (w) images (Sequence Name, SE T1; slice thickness of 4 mm; TR=891 and TE=15, flip angle of 90; matrix: 256×256; resolution: 1.9531×1.9531; number of slices: 135) through the entire lung were acquired with the patient breathing normally.

The MRI T1w and the CBCT images were transferred to a Philips Pinnacle treatment planning workstation (Philips Healthcare, Andover, MA) and the gross tumor volume (GTV) was contoured by a single radiation oncologist. The GTV volume was computed for the MRI T1w and CBCT images on the studies taken before the first fraction, on Day Three of treatment, and three months after completing SBRT.

## III. RESULTS

The GTV volumes of all the lesions contoured on CBCT and MRI before the first fraction and on Day Three of SBRT are shown in Table 2. On both CBCT and MRI, the volume differences between the image taken on Day Three of radiotherapy and the image taken before the first fraction are summarized in Table 3. The GTV volumes seen before SBRT and three months after completing SBRT are shown in Table 4.

**Table 2:** Target volume comparison of lung SBRT patients

*Patient No*.	*Tumor No*.	*Status*	*Cone‐beam CT (cm^3^)*	*MRI T1 WI (cm^3^)*
1	1	Before RT	9.46	9.32
		On 3rd Day	11.1	15.5
2	1	Before RT	15.43	17.07
		On 3rd Day	17.48	25.79
‐	2	Before RT	7.27	13.28
		On 3rd Day	6.41	16.38
3	1	Before RT	10.87	7.91
		On 3rd Day	14.26	11.6
4	1	Before RT	8.68	6.19
		On 3rd Day	12.8	8.54
5	1	Before RT	4.07	4.25
		On 3rd Day	5.76	4.45
‐	2	Before RT	7.06	4.76
		On 3rd Day	7.93	5.56
6	1	Before RT	5.48	5.89
		On 3rd Day	6.81	6.22
7	1	Before RT	12.21	10.92
		On 3rd Day	13.03	13.3
8	1	Before RT	13.82	16.56
		On 3rd Day	14.47	17.55
‐	2	Before RT	6.73	7.22
		On 3rd Day	9.26	8.77
9	1	Before RT	5.59	5.68
		On 3rd Day	6.95	6.61
10	1	Before RT	15.39	17.26
		On 3rd Day	16.95	17.46
Median size before RT			8.68	7.91
Median size on 3rd day			11.1	11.6

CT=computed tomography; RT=radiotherapy; SBRT=stereotactic body radiotherapy.

**Table 3:** Target volume difference between the first day and the third day of lung SBRT. Difference in percentage is shown in the parenthesis

*Patient No*.	*Tumor No*.	*Cone‐beam CT (cm^3^)*	*MRI T1WI (cm^3^)*
1	1	1.64 (117%)	6.18 (166%)
2	1	2.05 (113%)	8.72 (151%)
	2	−0.86(88%)	3.10 (123%)
3	1	3.39 (131%)	3.69 (147%)
4	1	4.12 (147%)	2.35 (138%)
5	1	1.69 (142%)	0.20 (105%)
	2	0.87 (112%)	0.80 (117%)
6	1	1.33 (124%)	0.33 (106%)
7	1	0.82 (107%)	2.38 (122%)
8	1	0.65 (105%)	0.99 (106%)
	2	2.53 (138%)	1.55 (121%)
9	1	1.36 (124%)	0.93 (116%)
10	1	1.56 (110%)	0.20 (101%)
Median difference		1.56 (117%)	1.55 (121%)

a CT=computed tomography; MRI=magnetic resonance imaging; SBRT=stereotactic body radiotherapy; WI=weight imaging.

**Table 4:** Target volume comparison on MRI T1WI, before and three months after radiotherapy of the SBRT patients (cm^3^). Difference in percentage is shown in parentheses

*Patient No*.	*Tumor No*.	*Status*	*MRI T1WI (cm^3^)*	*Difference in Tumor Volume (cm^3^)*
1	1	before RT	9.32	
		3 mos after RT	5.73	3.59 (61%)
2	1	before RT	17.07	
		3 mos after RT	4.33	12.74 (25%)
	2	before RT	13.28	
		3 mos after RT	0.86	12.42 (6%)
3	1	before RT	7.91	
		3 mos after RT	2.29	5.62 (29%)
4	1	before RT	6.19	
		3 mos after RT	2.33	3.86 (38%)
5	1	before RT	4.25	
		3 mos after RT	N/A	N/A
	2	before RT	4.76	
		3 mos after RT	N/A	N/A
6	1	before RT	5.89	
		3 mos after RT	1.03	4.86 (17%)
7	1	before RT	10.92	
		3 mos after RT	N/A	N/A
8	1	before RT	16.56	
		3 mos after RT	6.16	10.40 (37%)
	2	before RT	7.22	
		3 mos after RT	1.95	5.27 (27%)
9	1	before RT	5.68	
		3 mos after RT	5.05	0.63 (89%)
10	1	before RT	17.26	
		3 mos after RT	10.26	7.00 (59%)
Median size before RT			7.91	
Median size on the 3rd day			3.33	5.45 (29%)

a Mos=months; MRI=magnetic resonance imaging; SBRT=stereotactic body radiotherapy; RT=radiotherapy; WI=weight imaging.

The median tumor size on CBCT taken on Day One of SBRT was $8.68\;{\text{cm}}^{3}$ and on Day Three it was $11.10\;{\text{cm}}^{3}$. In all but one lesion, the tumor volume was larger on CBCT on Day Three of SBRT compared to the volume on the preradiotherapy image (median enlargement, $1.56\;{\text{cm}}^{3}$). The median tumor size on MRI T1w images taken before the start of radiotherapy was $7.91\;{\text{cm}}^{3}$. The median tumor size on SBRT was $11.60\;{\text{cm}}^{3}$ on Day Three. For all lesions, the tumor volume was larger on MRI T1w on Day Three of SBRT, compared to the preradiotherapy study (median enlargement, $1.55\;{\text{cm}}^{3}$). The GTV volume difference between the preradiotherapy MRI and that taken three months after completing SBRT is shown in Table 4. The patient with a smaller GTV on Day Three of radiotherapy, compared to the images taken before beginning radiotherapy, showed a strong artifact due to a postoperative clip on the CBCT (Fig. 2). The artifact wasn't detectable on MRI and did not affect the image quality (Fig. 3). However, the artifact might have caused the discrepancy in the results from the MRI and CBCT for this patient. One patient (Patient 5) was unable to undergo the last MRI because of deteriorating health due to multiple extrapulmonary metastases; another patient underwent MRI but the MRI images were not evaluable because of radiation pneumonitis (Patient 7). In all other patients, the GTV volumes were smaller on the images taken three months after completing SBRT, compared to the one taken before the start of SBRT (median shrinkage volume, $5.44\;{\text{cm}}^{3}$).

**Figure 1:**
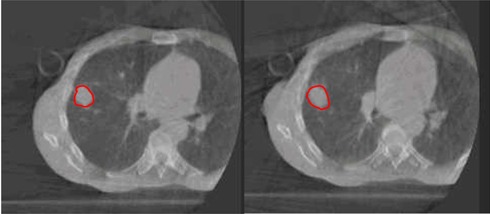
An example of GTV (red line) volume difference between the two CBCT scans for Patient 3: an image taken before SBRT (left) and an image taken after completing the 3rd fraction of SBRT (right).

**Figure 2:**
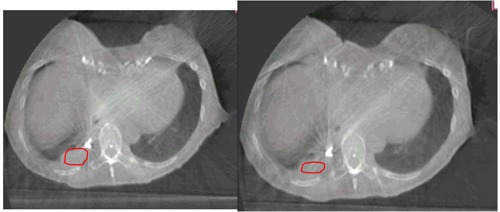
The CBCT image of the only lesion (Patient 2, tumor #2) for which the GTV (red line) volume difference was smaller on the image after the 3rd fraction compared to the image before the 1st fraction: image taken before SBRT (left) and image taken after treatment on Day 3 of SBRT (right). A prominent artifact, due to a metal clip, is detectable.

**Figure 3:**
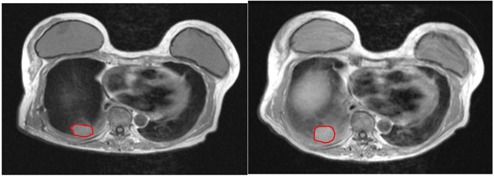
An example of GTV (red line) volume difference between the two scans in T1WI for Patient 2, tumor #2: image taken before SBRT (left) and image taken after treatment on Day Three of SBRT (right).

Figure 1 shows an example of GTV volume differences between the two scans on CBCT for Patient 3. Figure 2 demonstrates the only CBCT images where the GTV volume was smaller on the image taken on Day Three compared to the image taken on Day One (Patient 2, tumor #2). Figure 3 illustrates an example of GTV volume differences between the two scans on MRI T1w images for the second tumor in Patient 2.

## IV. DISCUSSION

Images were taken before and on Day Three after the beginning SBRT treatment. The reason for the increase in the GTV could be due to changes in breathing pattern between the planning and treatment scans, an increase in the size of the tumor, or both.

One published cause for changes in intrafractional breathing pattern is a change in the patient's relaxation.^(5)^ Although patients might be nervous during treatment planning, they may feel more comfortable at the time of treatment, thereby causing changes in breathing pattern; such an explanation is unlikely in our patient population as almost all patients showed an increase in GTV on Day Three of treatment using two different imaging modalities. In addition, it makes it difficult to attribute changes to breathing pattern alone since patients are taught to breathe normally for treatment. For all but the two patients without evaluable MRI images, the GTV size measured on the MRI taken three months after completing radiotherapy showed shrinkage of the GTV. Therefore, if there was an increase in tumor volume, it was a transient one. Huber et al.^(6)^ reported a retrospective study of their metastatic brain tumor patients treated with radiosurgery and found a transient enlargement on MRI taken six weeks after treatment. This enlargement was unrelated to tumor recurrence and, therefore, patients did not require further treatment. A similar phenomenon could occur in a lung and, as the surrounding lung tissue is softer than brain tissue, the resulting volume increase could be more obvious.

The present study has several limitations. First, because all images were taken during free breathing, the GTV size we contoured was not only the tumor volume, but also included volume caused by tumor motion. To evaluate the real tumor size over the course of SBRT, the patients would have to undergo breath‐holding CT and MRI scanning. Another limitation to our study is the potential low repeatability of the breathing cycle on some patients on both imaging studies (the CBCT and MRI examinations). Since the imaging was conducted under free‐breathing, the whole breathing cycle was unlikely to be correctly captured leading to potential underestimation of the ITV, in particular when the ratio of time spent in inspiration versus expiration deviated from unity.^(7)^

Guckenberger et al.^(8)^ reported a variability in the breathing motion of SBRT patients evaluated on their 1st, 2nd, and 3rd fractionations with CBCT. Of the 14 lesions reported by Guckenberger and colleagues, six showed enlargements, three mostly maintained their sizes, and five showed shrinkage of the breathing motion. The changes from intrafractional breathing‐induced tumor motion were evaluated based on the density distribution of the tumor in CBCT studies, which was done in the direction of the predominant tumor motion. By measuring only the predominant direction and using the density distribution, the Guckenberger study tried to focus more on the tumor motion than the actual tumor size. In our study, we contoured the tumor slice by slice (all directions were measured). Since the contouring was done manually, the faint density around the tumor, which is not detectable with the naked eye, was neglected. This might be one of the reasons our results showed a discrepancy to the study by Guckenberger et al.

## V. CONCLUSIONS

On CBCT and MRI, all but one patient (and only on the CBCT) showed a GTV enlargement with a median of $1.5\;{\text{cm}}^{3}$. Since these kinds of events are unpredictable, when treating with large fraction sizes such as with SBRT, tumor motion and tumor volume changes must be taken into account during treatment planning and image‐guided methods should be used.
